# Identification of Sex-Specific Transcriptome Responses to Polychlorinated Biphenyls (PCBs)

**DOI:** 10.1038/s41598-018-37449-y

**Published:** 2019-01-24

**Authors:** Almudena Espín-Pérez, Dennie G. A. J. Hebels, Hannu Kiviranta, Panu Rantakokko, Panagiotis Georgiadis, Maria Botsivali, Ingvar A. Bergdahl, Domenico Palli, Florentin Späth, Anders Johansson, Marc Chadeau-Hyam, Soterios A. Kyrtopoulos, Jos C. S. Kleinjans, Theo M. C. M. de Kok

**Affiliations:** 1 Department of Toxicogenomics, Maastricht University, Maastricht, The Netherlands; 2 MERLN Institute for Technology-inspired Regenerative Medicine, Maastricht University, Maastricht, The Netherlands; 3 Department of Health Protection, Chemicals and Health Unit, National Institute for Health and Welfare, Kuopio, Finland; 4 Institute of Biology, Medicinal Chemistry and Biotechnology, National Hellenic Research Foundation, Athens, Greece; 5 Department of Biobank Research, and Occupational and Environmental Medicine, Umeå University, Umeå, Sweden; 6 Molecular and Nutritional Epidemiology UnitI, ISPO Cancer Prevention and Research Institute, Florence, Italy; 7 Department of Epidemiology and Biostatistics, School of Public Health, Imperial College London, London, UK

## Abstract

PCBs are classified as xenoestrogens and carcinogens and their health risks may be sex-specific. To identify potential sex-specific responses to PCB-exposure we established gene expression profiles in a population study subdivided into females and males. Gene expression profiles were determined in a study population consisting of 512 subjects from the EnviroGenomarkers project, 217 subjects who developed lymphoma and 295 controls were selected in later life. We ran linear mixed models in order to find associations between gene expression and exposure to PCBs, while correcting for confounders, in particular distribution of white blood cells (WBC), as well as random effects. The analysis was subdivided according to sex and development of lymphoma in later life. The changes in gene expression as a result of exposure to the six studied PCB congeners were sex- and WBC type specific. The relatively large number of genes that are significantly associated with PCB-exposure in the female subpopulation already indicates different biological response mechanisms to PCBs between the two sexes. The interaction analysis between different PCBs and WBCs provides only a small overlap between sexes. In males, cancer-related pathways and in females immune system-related pathways are identified in association with PCBs and WBCs. Future lymphoma cases and controls for both sexes show different responses to the interaction of PCBs with WBCs, suggesting a role of the immune system in PCB-related cancer development.

## Introduction

Chemicals such as polychlorinated biphenyls (PCBs) are synthetic xenoestrogens with high solubility in fats and lipids and a long half-life, which causes an overall accumulation in the environment. PCBs were widely used in diverse industrial applications from the 1930s to the 1980s. Despite the prohibition of their production due to association with cancer risk and infertility^1^, PCBs can still be found in the environment. They have been classified by the International Agency for Research on Cancer (IARC) as human carcinogens based on epidemiological evidence^2^. They furthermore interfere in processes controlled by hormones and modify growth factors^3^.

It has also been suggested that exposure to xenoestrogens including PCBs might explain the sex-specific differences in cancer incidence that have been observed, where males showed to be more prone to cancer than females^4–6^.

Furthermore, PCB exposure has been associated with adverse effects on the immune system^7^ and immunosuppressive effects in unborn children^8,9^ and also in animal studies^10–14^. Exposure to environmental pollutants such as PCBs is known to induce changes in gene expression^4,15,16^. To investigate the molecular mechanisms involved in lymphoma risk in association with PCB exposure, we performed an analysis of genome-wide RNA expression established in blood samples of 649 subjects from the EnviroGenomarkers Project. This EU seventh framework project (http://www.envirogenomarkers.net/) aimed to develop ‘OMICS-based biomarkers’ for studying the role of environmental exposures in human health. The project applied different –omics technologies for the discovery and validation of novel biomarkers predictive of increased risks of chronic diseases in which the environment may play an important role, specifically breast cancer and Non Hodgkin’s lymphoma. Additionally, the Enivrogenomarkers project aimed to explore the association of such risk biomarkers with exposure to a number of high-priority or emerging environmental pollutants with carcinogenic, immunotoxic or hormone-like properties. The EnviroGenomarkers dataset presents some challenges in terms of the statistical analysis since it comprises two prospective cohorts from the Northern Sweden Health and Disease Study (NSHDS) and the European Prospective Investigation into Cancer and Nutrition (EPIC-Italy). In addition, significant batch effects need to be taken into account^17^. In order to address these challenges we analyzed the relationship between exposure to PCBs and gene expression using a linear mixed model (LMM), an increasingly prominent research tool in epidemiological data analysis because of its flexibility to correct for both fixed effects (such as demographic parameters) and technical variables (nuisance variables)^18^. We assessed the relationship between exposure to PCBs and gene expression using the LMM approach thereby adjusting for these potential confounders.

Since PCBs may affect the immune system, it is important to also have information on the distribution of white blood cells (WBC) in order to establish the effect of PCB exposure on human cancer risk. Information about the proportion of white blood cells (WBCs) is available from the EnviroGenomarkers dataset. The number of WBCs in the blood is adjusted in the LMM as a confounder but also as an interaction term together with PCBs due to its potential role in the causal path between the exposure and the gene expression.

**Figure 1:**
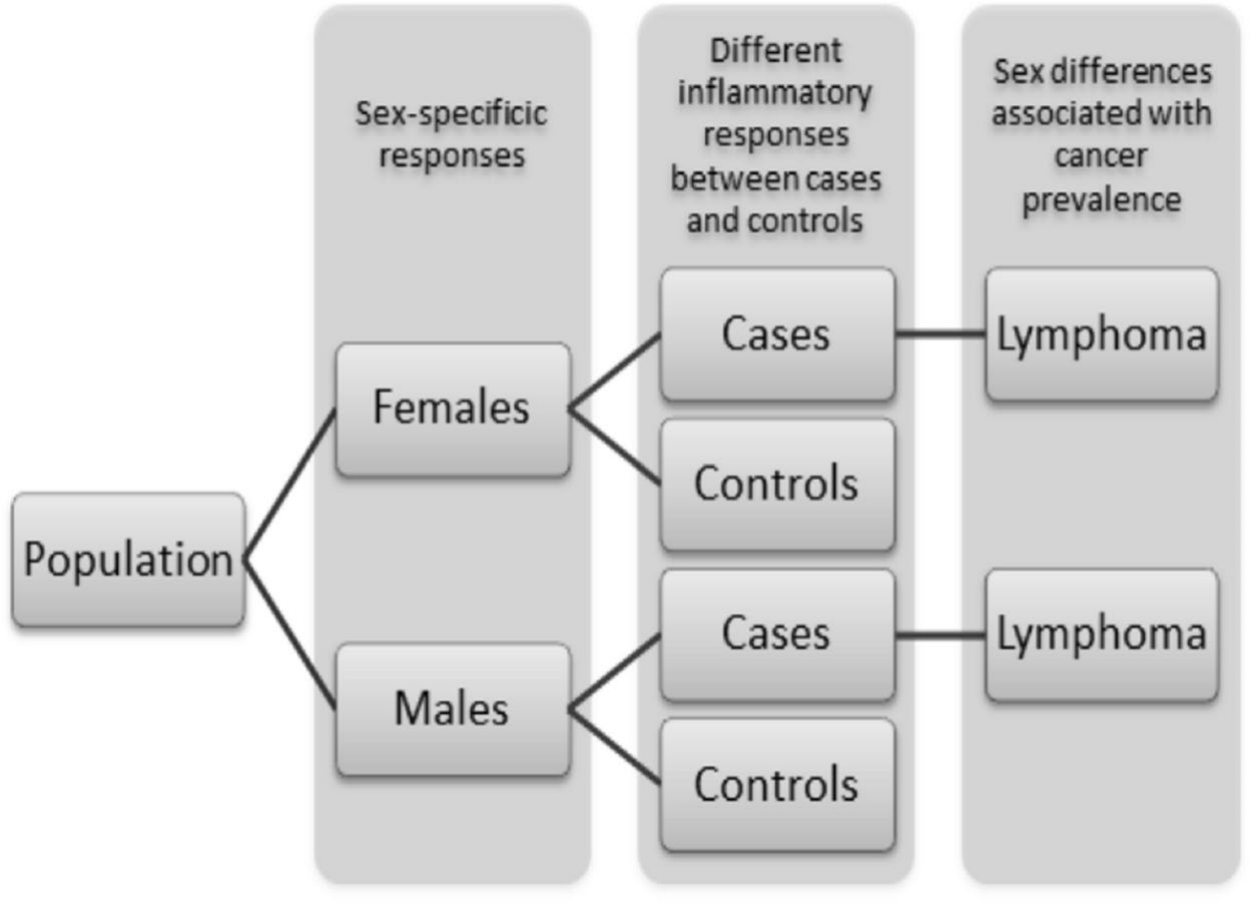
Stratification of the population for LMM analysis implementing PCB exposure and distribution of WBC, where each step address a different research question. *In addition to LMM implementing PCB exposure and distribution of WBC, LMM implementing PCB exposure alone has been performed.

The current study aims to establish the potential sex-specific gene expression changes induced by exposure to a mixture of PCBs under daily life circumstances by analyzing blood cell gene expression profiles in the population subdivided into females and males. Secondly, it sets out to assess the transcriptomic changes possibly mediated by immunotoxic effects associated with differences in the distribution of WBC. Finally, sex-specific differences in gene expression responses to PCB exposures in the subpopulation developing lymphoma in later life are identified (Fig. 1).

## Results

### Exposure level

**Figure 2:**
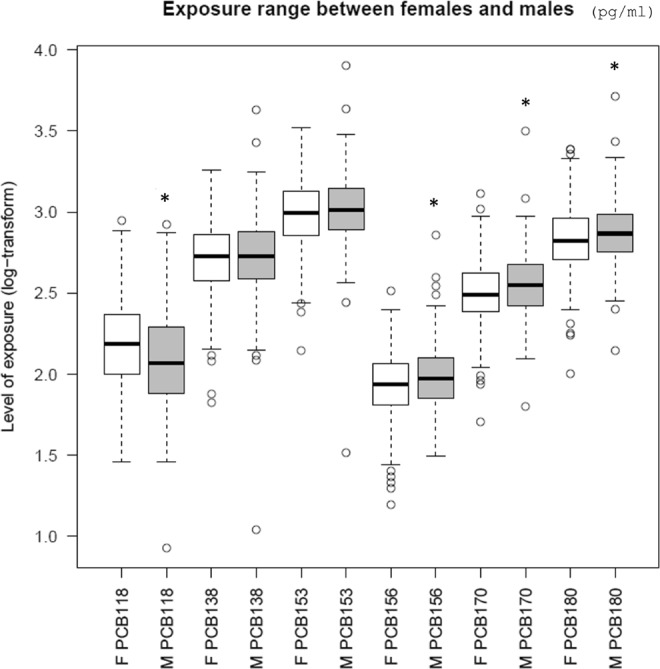
Range of PCB exposure for females (“F”, white boxplot) and males (“M”, grey boxplot). *p-value < 0.05 (Student’s t-test).

Exposure levels of PCB118, PCB156, PCB170 and PCB180 were significantly different between females and males (Student’s t-test, p-value < 0.05). Figure 2 presents individual exposure levels. Robust differences in the LMM signals obtained in the two sexes were still present after deleting those high/low exposed females that caused these statistically significant differences, thus suggesting a sex-specific transcriptional response to PCBs. Based on this and in view of the importance to evaluate a larger range of exposure, the entire female population was used for further analyses. Exposure levels of PCBs are not different when other variables such BMI are assessed.

### LMM for PCBs and gene expression: Sex-specific responses

Lists of transcripts associated with a p-value were generated from LMM analysis by comparing the levels of significance of the two models with and without a variable of interest. Afterwards an FDR correction (FDR cutoff of 0.05) was applied on the p-value following the Benjamini–Hochberg procedure^19^. The list of genes is available in the Excel document from Supplementary Material. As shown in the list representing the sum of transcript lists for individual PCBs from Table 1, the response to PCB exposure was only observed in the female population (mostly associated with the NSHDS cohort, as the cohorts did not differ regarding BMI or other registered variables). When a less strict FDR cutoff (FDR < 0.2) was applied, some genes appeared induced by PCB170 also in males. The top 3 deregulated pathways in females in which these genes were involved are mainly generic and relate to gene expression, metabolism of proteins, translation and cell cycle (Supplementary Table S2).

**Table 1:** Number of transcripts after linear mixed model analysis from the female and male population using only PCB as variable of interest.

Population	Variable of interest	FDR < 0.05
Females	PCB118	0
Females	PCB138	0
Females	PCB153	504
Females	PCB156	4824
Females	PCB170	3939
Females	PCB180	4163
Males	PCB118	0
Males	PCB138	0
Males	PCB153	0
Males	PCB156	0
Males	PCB170	0
Males	PCB180	0

The relatively large response to PCBs in the female population could potentially also be related to the average age of females 53.31 ± 7.81 years old, an age at which changes in the hormonal regulation occur involving for instance estrogen metabolism and menopausal processes. To test this hypothesis an analysis was conducted on 59 non-smoking females from the Italian cohort for whom age of menopause was registered. The LMM analysis specifically for PCB156, selected because it induces a large response (Table 1), controlling or not controlling for age of menopause as a confounder, gave different numbers of transcripts: with a FDR cutoff of 0.2, 53 hits for the not controlling approach and 134 hits for the controlling for age of menopause approach. The 53 genes were included within the 134 transcripts. The 81 additional hits that appeared after controlling for menopause age might be due to biological differences as a consequence of hormonal regulation. They were involved in pathways of DNA damage/telomere stress-induced senescence and in G1-to-S cell cycle control. Previous studies revealed associations between PCBs and breast cancer in postmenopausal women^20^ and lower immune function^21^. However, the low number of samples with postmenopausal information available could also cause overfitting and therefore produce false positives.

### LMM for analyzing the interaction between PCB exposure levels and distribution of WBC

We ran different LMM for the interaction between each individual PCB and WBC of the different individual WBC types in order to identify transcripts significantly associated with the combination of both PCBs and WBCs that are not found when looking at them individually. This means that the identified genes only respond to PCB exposure given a certain composition of the WBC population. The WBCs population itself might be also affected by the exposure, although this is not necessary required for the identification of these responding genes. The number of transcripts generated from each approach and appearing among men and women is presented in Supplementary Material Table S3. The list of genes is available in the Excel document from Supplementary Material. Interactions with B cell distribution appeared among both men and women, while interactions with natural killer cell and monocyte distribution appeared almost exclusively in women. The outcome of the pathway analysis is shown in Table 2, where cancer pathways specifically appear relatively stronger induced among males (8 out of 19 against 4 out of 18 among females).

**Table 2:** Pathways (q-value < 0.05) for interaction between PCBs and WBCs in all females and all males (transcriptomics approach, FDR < 0.05).

Names	Elements	Cancer related pathways
Females PCB153 Monocytes Females PCB156 Monocytes Females PCB170 Monocytes	Platelet degranulation	
	Response to elevated platelet cytosolic Ca2+	
	Platelet activation, signaling and aggregation	
	Hemostasis	
Females PCB118 B-cell Females PCB153 B-cell	Regulation of cytoplasmic and nuclear SMAD2/3 signaling	X
Females PCB118 B-cell	miR-targeted genes in muscle cell - TarBase	
	Signaling by Hedgehog	X
	HH-Ncore	
Females PCB153 B-cell	Regulation of nuclear SMAD2/3 signaling	X
	Antiviral mechanism by IFN-stimulated genes	
	Inositol Metabolism	
	Unfolded Protein Response (UPR)	
	tgf beta signaling pathway	X
	ISG15 antiviral mechanism	
Females PCB156 Monocytes	mcalpain and friends in cell motility	
Females PCB170 Monocytes	Formation of Fibrin Clot (Clotting Cascade)	
	ADP signalling through P2Y purinoceptor 12	
	Common Pathway of Fibrin Clot Formation	
Males PCB156 B-cell	Retrograde endocannabinoid signaling - Homo sapiens (human)	
Males PCB170 B-cell	BDNF signaling pathway	
Males PCB180 B-cell	Pathways in cancer - Homo sapiens (human)	X
Males PCB153 B-cell	Angiogenesis	X
Males PCB156 B-cell		
Males PCB156 B-cell	Ca2 + pathway	
	Proteoglycans in cancer - Homo sapiens (human)	X
	Circadian entrainment - Homo sapiens (human)	
	G-protein activation	X
	Opioid Signalling	
	G Protein Signaling Pathways	X
	GABAergic synapse - Homo sapiens (human)	
	Signaling by Wnt	X
	Morphine addiction - Homo sapiens (human)	
	Choline metabolism in cancer - Homo sapiens (human)	X
	Rap1 signaling pathway - Homo sapiens (human)	
	beta-catenin independent WNT signaling	X
Males PCB170 B-cell	Neural Crest Differentiation	
	Transport of glucose and other sugars, bile salts and organic acids, metal ions and amine compounds	
	Signaling pathways regulating pluripotency of stem cells - Homo sapiens (human)	

The overlap of hits with the list of genes associated with PCB exposure as retrieved from CTD (Comparative Toxicogenomics Database) was around 10%, showing a high number of hits (531 for females, 191 for males) that were previously not known to be associated with PCB exposure but that were significant when we studied the interaction between PCBs and WBCs. This complex interaction may explain why these genes have not been identified before. For females, platelet-related pathways were identified from the 90% remaining genes, whereas cancer-related pathways were found for males. None of these new pathways were overlapping with the gene list found associated with PCBs only. Figure S1 from Supplementary Material shows the gene-gene interactions between the novel genes found by analyzing the interaction of WBCs and PCBs (90%) and the genes reported in literature to be associated with PCBs (10%). The genes from the cancer-related pathways identified in Table 2 are highlighted in the network.

### LMM for evaluating the interaction between PCB exposure levels and distribution of WBC in control subjects and future lymphoma cases

The results from stratifying the population into healthy males and females that eventually developed lymphoma and healthy controls (males and females that remain healthy) showed very different numbers of transcripts (Table 3). The list of genes is available in the Excel document from Supplementary Material. There was no overlap between the lists of genes from the healthy and future disease population. Females showed highly significant pathways associated with the hits from the interaction PCBs and distribution of WBC only in the population of future cases and not in the controls (Supplementary Material Table S4). Males presented hits of cancer pathways associated with the interaction PCBs and B-cells in the future cases population and interaction PCBs and natural killers, CD4T and monocytes in the control population.

**Table 3:** Number of transcripts (FDR < 0.05) after LMM analysis after stratifying into controls and future cases for females and males using the interaction PCBs-WBCs.

Population	Cell type	PCB	Controls	Overlap	LY Future cases
Females	B cells	118	0	0	1055
Females	B cells	138	0	0	650
Females	B cells	153	0	0	563
Females	B cells	156	2	0	48
Females	B cells	170	2	0	14
Females	B cells	180	2	0	127
Females	CD8T	118	0	0	0
Females	CD8T	138	0	0	1
Females	CD8T	153	0	0	5
Females	CD8T	156	0	0	4
Females	CD8T	170	0	0	4
Females	CD8T	180	0	0	3
Females	CD4T	118	0	0	3121
Females	CD4T	138	0	0	2755
Females	CD4T	153	0	0	3004
Females	CD4T	156	0	0	3586
Females	CD4T	170	0	0	4326
Females	CD4T	180	0	0	5315
Females	NK	118	0	0	2
Females	NK	138	0	0	1
Females	NK	153	0	0	1
Females	NK	156	0	0	73
Females	NK	170	0	0	21
Females	NK	180	0	0	1
Females	Mono	118	0	0	723
Females	Mono	138	0	0	7445
Females	Mono	153	1	0	8373
Females	Mono	156	1	1	10120
Females	Mono	170	2	0	9352
Females	Mono	180	2	0	7944
Males	B cells	118	0	0	238
Males	B cells	138	0	0	195
Males	B cells	153	0	0	61
Males	B cells	156	0	0	28
Males	B cells	170	0	0	24
Males	B cells	180	0	0	14
Males	CD8T	118	1	0	0
Males	CD8T	138	1	0	0
Males	CD8T	153	1	0	0
Males	CD8T	156	1	0	0
Males	CD8T	170	1	0	0
Males	CD8T	180	1	0	0
Males	CD4T	118	1	0	0
Males	CD4T	138	0	0	0
Males	CD4T	153	0	0	0
Males	CD4T	156	0	0	0
Males	CD4T	170	26	0	0
Males	CD4T	180	34	0	0
Males	NK	118	0	0	0
Males	NK	138	0	0	0
Males	NK	153	1099	0	0
Males	NK	156	1227	0	1
Males	NK	170	3070	0	0
Males	NK	180	2621	0	0
Males	Mono	118	1	0	1
Males	Mono	138	0	0	2
Males	Mono	153	0	0	0
Males	Mono	156	21	0	0
Males	Mono	170	284	0	5
Males	Mono	180	182	0	1

The column “LY Future cases” in Table 3 corresponds to the subpopulation of future lymphoma cases. Despite the reduction of the population size and therefore statistical power, the fact that most of the highly significant genes found within each separate analysis were involved in common pathways showed that it is rather unlikely that these genes represented only false positives.

Male cases showed only high response from the interaction model from B-cells. The interaction involving the rest of WBCs did not result in a large response. There was only one common pathway present in both sexes (females PCB118, females PCB138, females PCB153 and males PCB138) which was ubiquitin-mediated proteolysis in human, a pathway potentially involved in cancer prevention^22^. TGF-beta receptor-related pathways were most frequent among females while in males cancer-related and neuronal-related pathways were predominant (Supplementary Material Table S4).

### Time of diagnosis

A highly significant but weak (low correlation coefficients) association between transcripts associated with PCB exposure identified in the PCBs and WBCs interaction LMM analysis and time of diagnosis was found. This implies that a higher or lower level of expression of transcripts is associated with a longer or shorter time to diagnosis. The correlation coefficients vary between −0.31 (p-value 5.05E-5) and 0.19 (p-value 0.011) for females, −0.16 (p-value 0.082) and 0.25 (p-value 0.009) for males. The list of genes from the females population negatively correlated (p-value < 0.05) with time of diagnosis showed TGF-beta receptor and cell cycle-related pathways (Supplementary Material Table S5). No significant association was found between the distribution of WBC and time of diagnosis.

The survival analyses identified a few features relevant to future lymphoma status and time to diagnosis (Supplementary Material Table S6). The list of pathways associated with these features is shown in Supplementary Material Table S7. The ROC curves generated shows an important overlap with the expected line, suggesting a random separation between future lymphoma disease and future healthy, instead of a powerful predictive model (Supplementary Material Figure S2).

### Lymphoma profile comparison

The genes corresponding to the top 5% over or under-expressed were compared to the list of genes identified by the LMM in association with PCB exposure. The significant hits from LMM did not show a substantial overlap with the top 5%, as shown in Supplementary Material Table S9, where it is displayed the number of genes and the overlap between the top 5% up/down-regulated genes from the profile and the significant genes from LMM (overlap expressed in percentages in the last four columns). This finding implies that the mechanisms found in association with PCBs might be different from the lymphoma disease specific signatures identified from the ArrayExpress database.

## Discussion

We investigated the impact of PCB exposure on gene expression profiles in a human population study by applying LMMs. In addition, we identified the effect of the interaction between individual PCBs and distribution of WBC. The small overlap observed between pathways affected by different individual PCBs suggests that also different biological mechanisms of action are activated by varying exposures (Supplementary Material Tables S3 and S4). In the same way, interaction between PCBs and different distribution of WBC resulted in different responses for each WBC, demonstrating the importance of considering individual congeners and distribution of WBC in data analyses. In addition, of the total number of 531 transcripts for females that were significantly associated with the interaction PCBs and WBCs, 90% of them have not previously been associated with PCB exposure. This implies that only by looking at PCBs induced-responses these novel targets cannot be identified. The interaction between PCB156 and B-cells resulted in a number of pathways directly related to cancer, especially for males. Deregulated cancer pathways among males were also observed in association with other PCBs (highlighted in the column “Cancer related pathways” from Table 2) and according to literature, lymphoma incidence among males is significantly higher than in females^22,23^. Platelet-related pathways were found in the female population in the interaction with PCBs and monocytes, which is not surprising in view of the fact that it has been reported earlier in literature associations between PCBs and cardiovascular disease^24^ and platelets and cardiovascular disease^25,26^. Figure S1 from Supplementary Material shows the gene network of the novel genes and the genes reported in literature, indicating the genes that are known to be associated with cancer disease from Table 2. There were two estrogen-related activity pathways for different PCBs in future lymphoma cases in females (Supplementary Material Table S4). In the male future cases subpopulation, the interaction between PCB138 and B-cells showed also a large effect on the pathway level while in females this occurred in the interaction between PCB118 and B-cells.

Individual PCB congeners have different physicochemical properties that lead to different mechanism of toxicity. Because of their structural differences, PCBs can be divided into dioxin-like and non dioxin-like compounds (co-planar and non-co-planar), although PCB118 can be considered as a mixed type inducer^27,28^. PCBs are known to cause genotoxicity, sperm DNA damage, gene mutation, epigenetic effects and changes in gene expression^2^. AhR, a ligand-activated transcription factor that regulates xenobiotic-metabolizing enzymes and suppression of apoptosis, is known to be activated by dioxin-like PCBs^2,29^. The AhR is involved in most of the dioxin-induced immune suppression processes as it was previously demonstrated in a human population study^8^. Non dioxin like PCBs, do not activate the AhR and are known to be neurotoxic and immunotoxic. However, these effects are only observed at much higher concentrations. In our study, the AhR was only found to respond significantly in the interaction models for PCBs and monocytes in females but in contrast to what it was reported in literature, AhR was modulated by both dioxin-like and non-dioxin-like PCBs. However, in the interaction models the association between the AhR expression levels and interaction of exposure and WBCs was negative. This might be explained by the fact that PeCB (a PCB congener) can induce apoptosis of monocytes, and therefore AhR could be activated because the interaction term is low, due to low levels of monocytes^30^. The AhR interacts with other transcription and signaling pathways such as MAPKs proteins and induces enzymes linked to bioactivation of xenobiotics such as CYPs, particularly the subfamily members of CYPs CYP1A and CYP2B^2^. MAPKs and several CYP isoforms such as CYP11B1 (which are not induced by AhR agonists) were found to be significant in our PCBs models only for females. We have seen induction of CYP11B1, an effect that might be related to the steroid 11b-hydroxyalse activity of aromatizing androstenedione to estrone. CYP11B1 has also previously been found to be associated with PCBs exposure^31^. NR3C and NR1D3 were also found to be associated with exposure to both dioxin-like and non-dioxin-like PCBs. Associations between exposure to dioxin-like PCBs and expression of C-Src, MAP Kinase, and CXCR4 have been described in literature^32^ but none of these genes is found in our study. In the same way, associations between NFKB1, STAT3, STAT5, GRIN2B, GRIA2, VEGF and FOXO3^33–36^ and non-dioxin-like PCBs are found in literature but not identified in our study. This lack of overlap might be a consequence of the species, tissue and dose differences between our study and those reported in the literature. However, the different pathways shown in the results in association with different PCBs suggest a variety of ways that each PCB interacts with genes and also independently of the classification (dioxin-like or non dioxin-like) relevant to the AhR-mediated activation^37,38^. Other toxic effects associated with PCBs are endocrine disruption by blocking the thyroid system functioning and neurotoxicity. We found in our study TSHB (Thyroid Stimulating Hormone Beta) in females as well as several neural signaling pathways in both females and males.

PCB exposure has been associated with a variety of cancers^39^. We found a high number of significant hits associated with the interaction of PCBs with WBCs that were not previously associated with PCB exposure. These new hits are involved in platelet-related pathways in females and cancer-related pathways in males. The platelet-derived growth factor has been linked with cancer previously^40,41^. Furthermore the combination of an immune challenge and PCB exposure may be a prerequisite for the development of lymphoma^42^. Immunosuppression and inflammation can be one of the adverse effects of exposure to environmental PCBs^43^ which eventually can lead to tumorigenesis^44^. This demonstrates the need for a detailed evaluation of the impact that different white blood cell types may have on gene expression responses to environmental exposures like PCBs. Therefore, we consider it of importance to take the influence of WBC distribution into account when assessing the biological effects induced by exposure to compounds like PCBs.

We found sex-specific differences in response to PCBs on the gene expression level. Small but significant differences in the exposure level of certain PCB congeners between men and women introduce some difficulties in the comparison of biological responses to PCBs. However, the strong signals that were found in the female population were still present after removing the high/low exposed individuals, which were responsible for the statistical difference in exposure as compared to the male exposure levels. This outcome together with the small variation in exposure level indicates that the large differences in response were likely due to biological differences between the sexes, and not to false positive findings. In addition, keeping the entire female population in the analysis has identified biological processes of relevance. Comparison of profiles of lymphoma future cases between males and females showed almost no overlap between the sexes on the pathway level (Supplementary Material Table S4). The disparity in responses to exposure between females and males were more likely due to a dissimilar biological response than to the small difference in exposure levels.

We assessed the direction of the expression of the genes involved in cancer-related pathways from males (Table 2). Seven out of eight genes that are involved in all of these pathways showed the same direction associated with PCB exposure as other studies comparing controls and certain cancer diseases including head, neck cancer, colorectal cancer, lung carcinoma, basal cell carcinoma and uterine carcinoma. These genes were GNG7^45^, AXIN2^46^, HIF1A^47^, FGF2^48^, PPAP2B^49^ and LAMC1^50^ and GNAO1^51^. GNG7, AXIN2, HIF1A, FGF2 and PPAP2B are found upregulated in our study in the same way as it is found in association with lymphoma disease according to literature^52–57^.

We observed that the interaction between PCB exposure and distribution of WBC had a different effect in future lymphoma cases and controls (Table 3). This finding suggests that the interaction term might play a role in the development of disease and therefore this information might be relevant for risk assessment and prevention of disease. However, we did not find strong evidence of a relationship between genes identified by LMM and time to diagnosis.

Natural killer cells are components from the innate immune system and they are the only type of cells that lack pro-tumorigenic function^44,58^. Moreover, tumor initiation by external factors influences the innate immune system to initiate cancer development^59,60^. In healthy male subjects, we found that the combined effect of PCBs and natural killers or monocytes (innate response) induce a larger response than the interaction between PCBs and CD4T, CD8T or B-cells (adaptive immune system). In future male cases, the response was the other way around, suggesting that healthy subjects might respond to PCBs by activating the innate immune system and subjects that are prone to development of lymphoma show an adaptive immune response.

Most of the genes identified in the lymphoma profile comparison (in the range of 77% to 100% with a median of 99.8%) are different from the genes identified in our study in association with PCBs, suggesting a different mechanism of action than indicated by the strongest signals in the lymphoma-specific pathways. These genes are involved in 150 unique pathways related to drug metabolism, cell cycle and immune response (Supplementary Material Table S7). Future cases had not being diagnosed with cancer at the moment of sampling. However, we cannot exclude that they are in an early stage of the disease.

## Conclusion

Results from LMM analysis showed sex-specific responses to PCBs. Males and females showed a significant transcriptional response to the interaction between PCB exposures and the distribution of WBC, indicating in particular the induction of cancer pathways among males and the involvement of the immune system among females. The relevant inflammatory responses in cancer development indicate the necessity of including the different WB cell types in the study in the context of risk assessment of environmental exposures such as PCBs.

## Methods

### The EnviroGenomarkers study population

Lifestyle factors and anthropometric measurements were registered at the time of sampling (demographics in Table S1 Supplementary material) and blood samples were frozen and stored for later analysis^61,62^. The quality of the biobank samples was previously evaluated, concluding amenability for OMICS analyses^63^. A follow-up of all recruited participants for a period of 2 to 17 years (5.96 ± 2.85) identified subjects that did not have a B-cell lymphoma at the moment of blood sampling but developed that in later life. These subjects are defined in this study as “future cases”, while not having a disease at the time of inclusion and sampling. These future cases were matched with controls based on sex, age, center of inclusion, fasting status and date of blood collection.

The EnviroGenomarkers project was approved by the Regional Ethical Review Board of the Umea Division of Medical Research and the Florence Health Unit Local Ethical Committee and all methods were carried out in accordance with the approved guidelines. All participants gave written informed consent.

### Laboratory analysis and pre-processing

Samples from EPIC-Italy were stored in cryostraws that were opened by cutting them with RNase-free tools on a stainless steel plate imbedded in a box of dry ice to prevent thawing during handling. Then the frozen buffy coat was pushed out with a thin stainless steel plunger directly into 1.2 mL of RNAlater (QIAGEN) solution. NSHDS samples from their cryovials were retrieved in a frozen state by making a small opening at the bottom of each vial using a hot plunger and pushing the sample out with another plunger. This material was immediately thawed in 1.2 mL of RNAlater (QIAGEN). RNA was isolated on the same day with the RiboPure™ Blood kit (Ambion, Austin, TX, USA) using the manufacturer’s miRNA isolation protocol.

Agilent 4 × 44 K human whole genome microarray analyses were conducted using our standard methodologies^63,64^. In brief, each RNA sample was reverse transcribed into cDNA and labeled with cyanine 3 prior to hybridization. Subsequently, slides were washed and scanned using the Agilent Technologies G2565CA DNA Microarray Scanner. The technical performance and quality of the microarrays was established by visual evaluation of the scan images before and after within- and between-array normalization (using LOESS and A-quantile, respectively). In order to ensure that the samples from the different biobanks were suitable for –omics analysis, the quality was extensively evaluated^63^. Probes that did not contain at least 75% of the maximum possible number of pixels were filtered out and imputation of the missing values was performed using the k nearest neighbors approach (k = 15, Euclidian metric). In total, 512 samples remained after quality assessment of the gene expression microarrays, removal of outliers and subjects with high incidence of missing values.

DNA was extracted from buffy coats. Samples were thawed on ice and DNA was isolated using the QIAamp Blood Mini Kit (QIAGEN), evaluating it spectrophotometrically and by agarose gel electrophoresis. Genome-wide analysis of DNA methylation using Infinium HumanMethylation450 BeadChips (Illumina, San Diego, CA, USA), which contain 485,764 probes (>99% with CpG dinucleotides), was conducted following the manufacturer’s recommendations^63^. Methylation module and an adaptation of the HumMeth27QCReport was used^65^. Within- and between-array normalization (linear LOESS and A-quantile, respectively) using Gene ARMADA^66^ and imputation of missing values using the *k* nearest neighbors approach (k = 15, Euclidian metric) was conducted.

PCBs 118, 138, 153, 156, 170, and 180 are abundant congeners that can describe the total exposure to a great extent. PCB congeners in serum samples were liquid-liquid extracted and analyzed with either HP 6890GC connected to Waters Autospec Ultima high resolution mass spectrometer (HRMS) or an Agilent 7000B Triple Quadrupole GC-MS/MS system^67^. Results of the reference material generated by both methods were collected and we concluded that they are comparable.

### Study of dependency between variables

The Variance Inflation Factor (R package “HH”)^68^ assesses the level of multicollinearity between effects. Variables that can be linearly predicted from others are not suitable to be analyzed in the same model. To evaluate this, we performed a test for PCB118, PCB138, PCB153, PCB156, PCB170, PCB180, cohort, sex, age, BMI, smoking status (smokers, former smokers and nonsmokers) and the distribution of WBC including natural killer cells, B-cells, monocytes, granulocytes and cytotoxic T-cells such as CD8T, CD4T. The distribution of WBC had been calculated from the DNA methylation data (Illumina Infinium HumanMethylation450 BeadChip platform) of the same subjects (measured in the context of the EnviroGenomarkers project), using a published algorithm from Houseman *et al*.^67^. This method quantifies the mixed composition of leukocytes using DNA methylation signatures. The cell mixture reconstruction method infers changes in the distribution of WBC using signatures from purified leukocyte samples and it has been validated using different datasets^69^.

The variance inflation factor test returned values below 4 for all the variables except for PCB exposure levels and granulocytes, meaning that the remaining confounders (cohort, sex, type of cancer, age, BMI, smoking status and distribution of WBC except for granulocytes) could be analyzed independently for an individual PCB in each LMM. The different PCBs were considered separately in the statistical analysis also due to the different mechanisms of action among them and between dioxin/non dioxin-like^70^.

### LMM analysis

LMMs were fitted in order to find associations between the level of PCB exposures and the level of gene expressions after pre-processing, thereby correcting for potential confounders and nuisance random variation. The general mixed model formula for subject *i* is represented by:

1 $${{\rm{Y}}}_{i}=\alpha +{ {\mathcal B} }_{{\rm{1}}}{{\rm{X}}}_{i}+{ {\mathcal B} }_{{\rm{2}}}{{\rm{FE}}}_{{\rm{i}}}+{{\rm{uA}}}_{i}+{\in }_{i}$$

where Yi represents the level of gene expression for each subject i, α the intercept of the model, Xi the independent variable of interest, FEi the vector of fixed effects for subject i, β1 and β2 regression coefficient of the variables, uAi the random effect due to nuisance variance and ∈ i is the residual error or difference between observed and calculated value by the model.

We applied this LMM in order to evaluate the total effect of the independent variable, e.g. the individual PCB exposures, on the gene expression. We controlled for the potential confounders cohort, sex, type of future cancer, age, BMI, smoking status as well as the individual WBC distribution values as fixed effects. The date of microarray scanning was added as a random effect. We corrected for false discovery rate using the Benjamini and Hochberg’s threshold at 5%.

### Stratification of the analysis

The population was divided into females and males for reasons mentioned earlier.

Power calculation for the different subpopulations was conducted using the R package “pwr” (“pwr.f2.test” function)^71^ to estimate for each sample size the number of predictors in order to reach enough statistical power and level of significance 0.05.

Further stratification based on cohort and smoking status has been considered for females and males. We observed differences among groups in terms of number of hits but due to the large reduction of sample size, it was decided to correct for the variables “cohort” and “smoking status” in the whole study population.

### Mixed models implementing exposure and distribution of WBC

Several approaches using LMMs were explored in order to study the impact of PCBs on gene expression. The first comprises the analysis of individual PCBs, accounting for WBC distribution as confounders together with the other variables. This approach was applied to the whole population as well as to females and males separately.

In view of the potential influence of the presence of different blood cell types on PCB-induced gene expression profiles, a second approach implementing WBC distributionwas applied. Here, the variable of interest is presented by the interaction term between the individual PCBs and the different cell types:

2 $${{\rm{Y}}}_{i}=\alpha +{\beta }_{{\rm{1}}}{{\rm{PCB}}}_{i}+{\beta }_{{\rm{2}}}{{\rm{WBC}}}_{i}+{\beta }_{{\rm{3}}}{{\rm{PCB}}}_{i}{{\rm{xWBC}}}_{i}+{\beta }_{{\rm{4}}}{{\rm{FE}}}_{i}+{{\rm{uA}}}_{i}+{\in }_{I}$$

### Initial biological interpretation

In order to assess the additional biological information provided by analyzing the interaction of PCBs and WBCs, we compared the list of significant hits for males and females separately with a list of genes known to be associated with PCB exposure by exploiting the Comparative Toxicogenomics Database (http://ctdbase.org/)^72^.

### Pathway analyses

The lists of gene expressions significantly associated with PCB exposure were used to perform pathway analysis by means of ConsensusPathDB^73^. The analysis was executed using the default parameters. Each input list of genes was compared against a background list created specifically for this analysis containing the total list of genes after the preprocessing filtering.

### Lymphoma profile comparison

In order to relate transcriptomic responses to PCB exposure to the future disease status, from the ArrayExpress database (https://www.ebi.ac.uk/arrayexpress/), 34 microarray data sets based on blood samples from T-cell lymphoma patients were downloaded, of which 14 corresponded to females and 20 to males. Due to difficulties in finding more suitable databases for this study, T-cell lymphoma has to be used as a proxy for all lymphoma subcases. Additionally, another 100 microarray data sets from blood samples of healthy volunteers (50% females and 50% males) were used as controls^4^. Fold changes and level of significance were calculated using the “Limma” R package^74^. In view of the large number of differentially expressed genes obtained (two thirds of the total genes: cutoff fold change |1.5|, adjusted p-value 0.05) and in order to highlight specific lymphoma-related genes, only significant genes from the top 5% and the bottom 5% in fold change were selected for creating the lymphoma signature.

### Time to diagnosis

The time from sampling to diagnosis of disease in future cases might give some information about the development of disease. We therefore calculated the correlation between the expression after batch correction using LMM of significant genes and the time of diagnosis of lymphoma for females and males. We also studied the correlation between the level of WBCs and time of diagnosis.

Survival models in combination with elastic net were run in order to assess the predictive power of our list of transcripts to classify future lymphoma disease. All the unique transcripts that were significant were selected from all the statistical models from Table 3 (FDR 0.05), separately for females (12,938 transcripts in total) and males (3,767 transcripts in total). PCBs levels and the distribution of WBCs were added as additional features. Then, we conducted survival analyses using the R function “glmnet”, with “family” as “cox” from the R packages “survival” and “glmnet”. The survival analyses were modelled using status (future disease or future healthy) and time to diagnosis as input.

ROC curves were also generated using the R packages “caret” and “ROCR”. The same features as the survival analyses from above were used to apply elastic net, dividing the dataset into train dataset (70% of the original dataset) and test dataset (30% of the original dataset).

## Supplementary information

Supplementary Material

Supplementary Material

## Data Availability

The data set from the EPIC-Italy cohort supporting the results of this article is available in the Array repository, accession ID E-MTAB-7273. The data set from the NSHDS cohort is available upon request. Requests for the individual-level data can be made to the Department of Biobank Research, Umeå University (http://www.biobank.umu.se/biobank/nshds/), and will be subject to ethical review and assessment by a panel of scientists. Individual-level data cannot be made publicly available due to legal restrictions imposed by the Swedish Data Protection Authority. All relevant aggregated data are presented in the article. Contact details: Ingvar Bergdahl (ingvar.bergdahl@umu.se) or Åsa Ågren (asa.agren@umu.se).
