# Supplementary material for: Identification of Sex-Specific Transcriptome Responses to Polychlorinated Biphenyls (PCBs)
Source: Sci Rep. 2019 Jan 24;9:746. doi: 10.1038/s41598-018-37449-y (PMC6346099; doi:10.1038/s41598-018-37449-y)
Supplement: Supplementary file 1 — Supplementary Material [file 41598_2018_37449_MOESM1_ESM.pdf]

Supplemental Material

Identification of Sex-Specific Transcriptome Responses to Polychlorinated Biphenyls (PCBs)

Almudena Espín-Pérez[1], Dennie G. A. J. Hebels[1,2], Hannu Kiviranta[3], Panu Rantakokko[3], Panagiotis Georgiadis[4], Maria Botsivali[4], Ingvar A. Bergdahl[5], Domenico Palli[6], Florentin Späth[5], Anders Johansson[5], Marc Chadeau-Hyam[7], Soterios A. Kyrtopoulos[4], Jos C. S. Kleinjans[1], Theo M. C. M. de Kok[1]

[1]Department of Toxicogenomics, Maastricht University, Maastricht, The Netherlands

[2]MERLN Institute for Technology-inspired Regenerative Medicine, Maastricht University, Maastricht, The Netherlands

[3]Department of Health Protection, Chemicals and Health Unit, National Institute for Health and Welfare, Kuopio, Finland

[4]Institute of Biology, Medicinal Chemistry and Biotechnology, National Hellenic Research Foundation, Athens, Greece.

[5]Department of Biobank Research, and Occupational and Environmental Medicine, Umeå University, Umeå, Sweden

[6]Molecular and Nutritional Epidemiology Unit, Institute for Cancer Research and Prevention in Firenze, Italy

[7]Department of Epidemiology and Biostatistics, School of Public Health, Imperial College London, London, UK

Table S1. Demographics of the study population.

A) Number of subjects per subgroup

Characteristics		Control (*N=295*)	Future Lymphoma cases (N=217)
Age		52.10±7.83	52.34±8.22
Gender	Female	184	104
	Male	111	113
Cohort	Italy	118	75
	Sweden	177	142
BMI (kg/m2)		25.82±3.67	26.11±3.42
Smoking Status	Current	67	46
	Former	84	77
	Never	144	94

B) PCBs levels in women with menopause status and no menopause status

PCBs	Menopause	No menopause	P-value T-test (Menopause vs No menopause)
PCB118	182.03±124.67	190.03±126.83	0.25
PCB138	573.31±307.06	627.20±275.98	0.48
PCB153	1090.38±556.57	1137.75±461.24	0.93
PCB156	94.30±46.59	100.39±39.17	0.83
PCB170	344.9±168.38	367.68±147.79	0.68
PCB180	753.10±397.48	760.91±325.53	0.39

C) Correlation between PCBs and BMI in females and males

PCBs	Females	P-value correlation	Males	P-value correlation
PCB118	0.200	0.86	0.139	0.86
PCB138	0.082	0.34	0.104	0.32
PCB153	0.039	0.47	0.054	0.45
PCB156	0.002	0.40	-0.016	0.37
PCB170	-0.029	0.34	-0.033	0.33
PCB180	-0.046	0.39	-0.067	0.36

Table S2. Pathway analysis from the female's population gene list (transcriptomics approach, FDR<0.05) using only PCB as variable of interest. Pathway analysis from the sum of genes for all PCBs together (q-value < 0.05)

q-value	Pathway
1.90E-11	Gene Expression
2.02E-11	Metabolism of proteins
2.13E-10	Translation
5.53E-10	Eukaryotic Translation Elongation
5.53E-10	Eukaryotic Translation Termination
5.85E-10	Nonsense Mediated Decay (NMD) enhanced by the Exon Junction Complex (EJC)
5.85E-10	Nonsense-Mediated Decay (NMD)
6.02E-10	L13a-mediated translational silencing of Ceruloplasmin expression
6.02E-10	3, -UTR-mediated translational regulation
9.08E-10	GTP hydrolysis and joining of the 60S ribosomal subunit
1.02E-09	Nonsense Mediated Decay (NMD) independent of the Exon Junction Complex (EJC)
1.29E-09	Ribosome - Homo sapiens (human)
1.29E-09	Cap-dependent Translation Initiation
1.29E-09	Eukaryotic Translation Initiation
1.63E-09	Peptide chain elongation
2.46E-09	Formation of a pool of free 40S subunits
3.54E-09	SRP-dependent cotranslational protein targeting to membrane
1.82E-08	Cytoplasmic Ribosomal Proteins
4.12E-06	HIV Infection
3.45E-05	Cell Cycle
5.54E-05	mRNA Processing
6.46E-05	Organelle biogenesis and maintenance
0.000139	Translation initiation complex formation
0.000139	Ribosomal scanning and start codon recognition
0.000215	Activation of the mRNA upon binding of the cap-binding complex and eIFs, and subsequent binding to 43S
0.000215	Host Interactions of HIV factors
0.000233	Transcription
0.000254	HIV Life Cycle
0.000281	Mitochondrial translation termination
0.000281	Processing of Capped Intron-Containing Pre-mRNA
0.000317	Chromatin modifying enzymes
0.000317	Chromatin organization
0.000464	Cell Cycle, Mitotic
0.000533	RNA Polymerase I, RNA Polymerase III, and Mitochondrial Transcription
0.000547	Late Phase of HIV Life Cycle
0.000597	Electron Transport Chain
0.000692	Respiratory electron transport, ATP synthesis by chemiosmotic coupling, and heat production by uncoupling proteins.
0.000719	Formation of the ternary complex, and subsequently, the 43S complex

0.000727	The citric acid (TCA) cycle and respiratory electron transport
0.000987	Respiratory electron transport
0.001424	Mitochondrial translation
0.001531	Polycystic Kidney Disease Pathway
0.001531	Mitochondrial translation initiation
0.001531	Mitochondrial translation elongation
0.001611	Transcriptional regulation by small RNAs
0.001739	Cellular responses to stress
0.005518	Infectious disease
0.005763	Senescence-Associated Secretory Phenotype (SASP)
0.005763	Mismatch repair (MMR) directed by MSH2:MSH6 (MutSalpha)
0.005763	CD4 T cell receptor signaling-NFkB cascade
0.007134	Processive synthesis on the C-strand of the telomere
0.007147	DNA Repair
0.007301	Spliceosome - Homo sapiens (human)
0.007634	RNA Polymerase I Transcription
0.007634	Adaptive Immune System
0.008602	HATs acetylate histones
0.008986	CD4 T cell receptor signaling-JNK cascade
0.00901	Transcription-coupled NER (TC-NER)
0.009984	RNA Polymerase I Promoter Clearance
0.010777	role of mef2d in t-cell apoptosis
0.010777	mRNA Splicing - Major Pathway
0.010777	mRNA Splicing
0.01099	Mismatch Repair
0.01119	SUMOylation
0.012863	Regulatory RNA pathways
0.01544	TCR signaling in naive;ve CD4+ T cells
0.015705	Nucleotide Excision Repair
0.016219	Transport of Mature Transcript to Cytoplasm
0.017202	CD4 T cell receptor signaling-ERK cascade
0.021223	Processive synthesis on the lagging strand
0.02474	Dual hijack model of Vif in HIV infection
0.027216	RMTs methylate histone arginines
0.027216	RNA Polymerase I Chain Elongation
0.027216	G2/M Checkpoints
0.027509	CD4 T cell receptor signaling
0.027585	G2/M DNA damage checkpoint
0.027585	Biosynthesis of the N-glycan precursor (dolichol lipid-linked oligosaccharide, LLO) and transfer to a nascent protein
0.027585	Mismatch repair (MMR) directed by MSH2:MSH3 (MutSbeta)
0.028013	Antigen processing: Ubiquitination & Proteasome degradation
0.028159	Post-translational protein modification
0.02822	Mismatch repair - Homo sapiens (human)
0.029665	Transport of Mature mRNA derived from an Intron-Containing Transcript

0.029665	Huntington,s disease - Homo sapiens (human)
0.031392	Global Genomic NER (GG-NER)
0.031392	bcr signaling pathway
0.031392	SUMOylation of DNA damage response and repair proteins
0.031392	SUMO E3 ligases SUMOylate target proteins
0.034392	Alzheimer disease - Homo sapiens (human)
0.035958	Parkinson disease - Homo sapiens (human)
0.041422	IL2-mediated signaling events
0.041995	Mitotic Prophase
0.044009	Fc-epsilon receptor I signaling in mast cells
0.044009	Nuclear import of Rev protein
0.045593	Removal of the Flap Intermediate from the C-strand
0.046677	Telomere Maintenance
0.047575	Epigenetic regulation of gene expression

Table S3. Number of transcripts after linear mixed model analysis from the female (2A) and male (2B) population using the interaction PCB and WBCs as variable of interest

A) Females

Population	Cell type	PCB	FDR<0.05
Females	B cells	118	203
Females	B cells	138	0
Females	B cells	153	63
Females	B cells	156	5
Females	B cells	170	7
Females	B cells	180	5
Females	CD8T	118	0
Females	CD8T	138	0
Females	CD8T	153	0
Females	CD8T	156	0
Females	CD8T	170	0
Females	CD8T	180	0
Females	CD4T	118	1
Females	CD4T	138	0
Females	CD4T	153	0
Females	CD4T	156	2
Females	CD4T	170	1
Females	CD4T	180	2
Females	NK	118	58
Females	NK	138	0
Females	NK	153	1
Females	NK	156	0
Females	NK	170	0
Females	NK	180	0

Females	Mono	118	0
Females	Mono	138	0
Females	Mono	153	200
Females	Mono	156	448
Females	Mono	170	200
Females	Mono	180	64

B) Males

**Population**	**Cell type**	**PCB**	**FDR<0.05**
Males	B cells	118	226
Males	B cells	138	146
Males	B cells	153	62
Males	B cells	156	44
Males	B cells	170	31
Males	B cells	180	29
Males	CD8T	118	0
Males	CD8T	138	1
Males	CD8T	153	0
Males	CD8T	156	0
Males	CD8T	170	0
Males	CD8T	180	0
Males	CD4T	118	0
Males	CD4T	138	0
Males	CD4T	153	0
Males	CD4T	156	0
Males	CD4T	170	2
Males	CD4T	180	1
Males	NK	118	0
Males	NK	138	0
Males	NK	153	0
Males	NK	156	0
Males	NK	170	0
Males	NK	180	0
Males	Mono	118	0
Males	Mono	138	0
Males	Mono	153	0
Males	Mono	156	0
Males	Mono	170	8
Males	Mono	180	0

Figure S1. Cytoscape figure of hits significantly associated with the interaction between WBCs and PCBs for females (A) and males (B). *Can be found at:*
http://web.tgx.unimaas.nl/shared_files/aespin/EGM/females.jpeg
http://web.tgx.unimaas.nl/shared_files/aespin/EGM/males.jpeg

A)

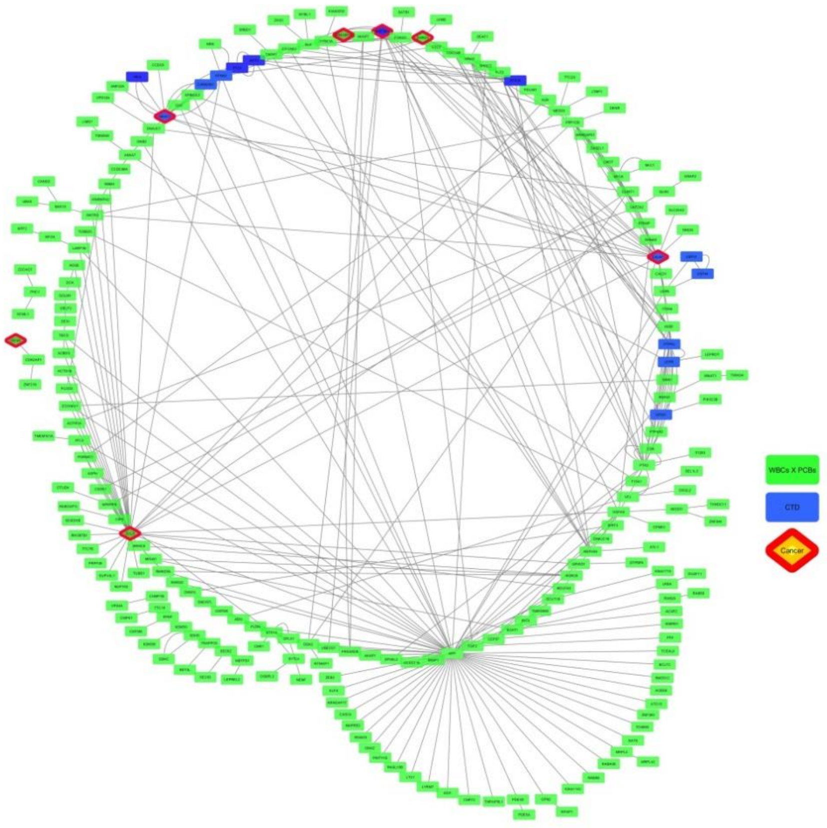

B)

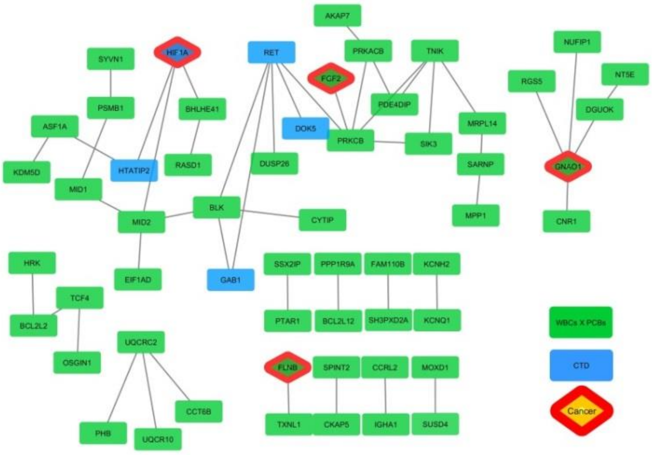

Table S4. Pathways (q-value<0.05) in only lymphoma cases: females and males comparison (transcriptomics approach, FDR<0.05).

Names	Total	Pathways
Females PCB118 B-cell Females PCB138 B-cell Females PCB153 B-cell Males PCB138 B-cell	1	Ubiquitin mediated proteolysis - Homo sapiens (human)
Females PCB118 B-cell Females PCB138 B-cell Females PCB153 B-cell Females PCB180 B-cell	11	TGF-beta receptor signaling activates SMADs
		FoxO signaling pathway - Homo sapiens (human)
		tgf beta signaling pathway
		Regulation of cytoplasmic and nuclear SMAD2/3 signaling
		Signaling by TGF-beta Receptor Complex
		Downregulation of TGF-beta receptor signaling
		Regulation of nuclear SMAD2/3 signaling
		ctcf: first multivalent nuclear factor
		Transcriptional regulation of pluripotent stem cells
		TGF_beta_Receptor
		nfkb activation by nontypeable hemophilus influenzae
Females PCB118 B-cell Females PCB138 B-cell Females PCB153 B-cell	21	miR-targeted genes in squamous cell - TarBase
		Endocytosis - Homo sapiens (human)
		HDMs demethylate histones
		Chromatin organization
		IRE1alpha activates chaperones
		Class I MHC mediated antigen processing & presentation
		miR-targeted genes in muscle cell - TarBase
		TGF-Ncore
		EGFR1
		XBP1(S) activates chaperone genes
		TGF beta Signaling Pathway
		TGF Beta Signaling Pathway
		pdgf signaling pathway
		ErbB1 downstream signaling
		Chromatin modifying enzymes
		miR-targeted genes in epithelium - TarBase
		Cell Cycle
		Unfolded Protein Response (UPR)
		T-Cell Receptor and Co-stimulatory Signaling
		miR-targeted genes in lymphocytes - TarBase
		Circadian Clock
Females PCB118 B-cell Females PCB138 B-cell Females PCB180 B-cell	1	Validated targets of C-MYC transcriptional repression
Males PCB156 B-cell	2	Pathways in cancer - Homo sapiens (human)

Males PCB170 B-cell Males PCB180 B-cell		Retrograde endocannabinoid signaling - Homo sapiens (human)
Females PCB153 B-cell Females PCB156 B-cell Females PCB180 B-cell	2	ISG15 antiviral mechanism
		Antiviral mechanism by IFN-stimulated genes
Females PCB138 B-cell Females PCB153 B-cell	8	Neurotransmitter uptake and Metabolism In Glial Cells
		Validated nuclear estrogen receptor alpha network
		Astrocytic Glutamate-Glutamine Uptake And Metabolism
		LIF signaling
		Endoderm Differentiation
		ER-Phagosome pathway
		Gene Expression
		mRNA Processing
Females PCB118 B-cell Females PCB138 B-cell	4	Adaptive Immune System
		Chronic myeloid leukemia - Homo sapiens (human)
		AndrogenReceptor
		Signal regulatory protein (SIRP) family interactions
Males PCB156 B-cell Males PCB170 B-cell	2	Neural Crest Differentiation
		Signaling pathways regulating pluripotency of stem cells - Homo sapiens (human)
Females PCB153 B-cell Females PCB180 B-cell	1	Regulation of autophagy - Homo sapiens (human)
Females PCB118 B-cell Females PCB153 B-cell	5	miR-targeted genes in leukocytes - TarBase
		Antigen processing: Ubiquitination & Proteasome degradation
		Validated targets of C-MYC transcriptional activation
		EGF-Core
		protein ubiquitylation
Females PCB156 B-cell Females PCB180 B-cell	2	Inositol Metabolism
		Interferon Signaling
Females PCB118 B-cell Females PCB180 B-cell	1	Downregulation of SMAD2/3:SMAD4 transcriptional activity
Males PCB138 B-cell	29	Vpu mediated degradation of CD4
		Regulation of mitotic cell cycle
		GLI3 is processed to GLI3R by the proteasome
		IL1
		SCF(Skp2)-mediated degradation of p27/p21
		Regulation of APC/C activators between G1/S and early anaphase
		Oxygen-dependent proline hydroxylation of Hypoxia-inducible Factor Alpha
		APC/C-mediated degradation of cell cycle proteins
		Degradation of GLI2 by the proteasome
		Signaling by Interleukins
		TNFalpha

		Signaling by Hedgehog
		Hh mutants that don,t undergo autocatalytic processing are degraded by ERAD
		Cytokine Signaling in Immune system
		Small Ligand GPCRs
		EGF-Ncore
		Hh mutants abrogate ligand secretion
		degradation of AXIN
		Angiogenesis
		Autodegradation of Cdh1 by Cdh1:APC/C
		NOTCH1 Intracellular Domain Regulates Transcription
		Hedgehog ,off, state
		JNK (c-Jun kinases) phosphorylation and activation mediated by activated human TAK1
		Degradation of beta catenin
		Regulation of nuclear beta catenin signaling and target gene transcription
		Interleukin-1 signaling
		Degradation of GLI1 by the proteasome
		SCF-beta-TrCP mediated degradation of Emi1
		metabolism of anandamide an endogenous cannabinoid
Females PCB138 B-cell	10	Retinoblastoma (RB) in Cancer
		G2/M Transition
		Cell cycle - Homo sapiens (human)
		Signaling events mediated by HDAC Class I
		Role of Calcineurin-dependent NFAT signaling in lymphocytes
		Canonical NF-kappaB pathway
		HuR stabilizes mRNA
		Regulation of Telomerase
		Cell Cycle, Mitotic
		Mitotic G2-G2/M phases
Males PCB170 B-cell	1	Hippo signaling pathway - Homo sapiens (human)
Females PCB153 B-cell	7	Gastric cancer network 2
		mTOR signaling pathway
		p38 mapk signaling pathway
		mRNA surveillance pathway - Homo sapiens (human)
		hypoxia-inducible factor in the cardivascular system
		mRNA Splicing - Major Pathway
		mRNA Splicing
Males PCB156 B-cell	2	BDNF signaling pathway
		G Protein Signaling Pathways
Females PCB156 B-cell	9	IL-1 p38
		EPHB forward signaling
		TLR JNK
		TLR p38

		IL-1 JNK
		Inositol phosphate metabolism - Homo sapiens (human)
		superpathway of inositol phosphate compounds
		IL-1 NFkB
		TLR NFkB
Males PCB180 B-cell	3	Signaling by Wnt
		GPCR ligand binding
		G alpha (i) signalling events
Females PCB180 B-cell	12	Hedgehog
		Signaling by Activin
		JAK STAT pathway and regulation
		NOD1/2 Signaling Pathway
		Gemcitabine Metabolism Pathway
		Gemcitabine Pathway, Pharmacodynamics
		TCR signaling
		Tuberculosis - Homo sapiens (human)
		SMAD2/SMAD3:SMAD4 heterotrimer regulates transcription
		Gemcitabine Action Pathway
		Signaling by NODAL
		Extracellular vesicle-mediated signaling in recipient cells
Females PCB118 B-cell	57	Protein processing in endoplasmic reticulum - Homo sapiens (human)
		p53 signaling pathway - Homo sapiens (human)
		igf-1 signaling pathway
		Transcriptional activity of SMAD2/SMAD3:SMAD4 heterotrimer
		Formation of apoptosome
		CXCR4-mediated signaling events
		SMAD2/3 MH2 Domain Mutants in Cancer
		Proteoglycans in cancer - Homo sapiens (human)
		Androgen receptor signaling pathway
		overview of telomerase rna component gene hterc transcriptional regulation
		RhoA signaling pathway
		DNA Damage Response
		RNA Polymerase II Transcription Termination
		Transcriptional misregulation in cancer - Homo sapiens (human)
		Loss of Function of SMAD2/3 in Cancer
		Spermidine and Spermine Biosynthesis
		Coregulation of Androgen receptor activity
		mRNA 3,-end processing
		PDGFR-beta signaling pathway
		Betaine Metabolism
		Plasma membrane estrogen receptor signaling
		EGF
		C-MYB transcription factor network

		B cell receptor signaling
		Notch-mediated HES/HEY network
		BCR signaling pathway
		Post-Elongation Processing of the Transcript
		Diseases of signal transduction
		Signaling by FGFR1 fusion mutants
		S-adenosyl-L-methionine biosynthesis
		Adherens junction - Homo sapiens (human)
		SMAD2/3 Phosphorylation Motif Mutants in Cancer
		bcr signaling pathway
		IL5
		Signaling by FGFR1 mutants
		Signaling by the B Cell Receptor (BCR)
		EGF-EGFR Signaling Pathway
		regulation of ck1/cdk5 by type 1 glutamate receptors
		TCR
		DARPP-32 events
		Interactome of polycomb repressive complex 2 (PRC2)
		signaling pathway from g-protein families
		DNA Damage Response (only ATM dependent)
		SHC-related events triggered by IGF1R
		Herpes simplex infection - Homo sapiens (human)
		IGF-Ncore
		Signaling by FGFR1 in disease
		Cleavage of Growing Transcript in the Termination Region
		Hepatitis B - Homo sapiens (human)
		B cell receptor signaling pathway - Homo sapiens (human)
		Class I PI3K signaling events
		TGF-beta receptor signaling
		BCR
		Signaling by TGF-beta Receptor Complex in Cancer
		RNA Polymerase II Transcription
		Post-Elongation Processing of Intron-Containing pre-mRNA
		Immune System

Table S5. Pathway from the genes significant in the females future lymphoma population that correlates negatively with time of diagnosis (p-value<0.05).

q-value	pathway
0.004261	Signaling by TGF-beta Receptor Complex
0.013531	TGF-beta receptor signaling activates SMADs
0.013531	Circadian Clock
0.025237	RNA degradation - Homo sapiens (human)
0.040462	regulation of ck1/cdk5 by type 1 glutamate receptors
0.040462	Downregulation of TGF-beta receptor signaling
0.040462	DARPP-32 events
0.040462	Regulation of cholesterol biosynthesis by SREBP (SREBF)
0.040462	Oxytocin signaling pathway - Homo sapiens (human)
0.040462	Intracellular Signalling Through Histamine H2 Receptor and Histamine
0.040462	Excitatory Neural Signalling Through 5-HTR 6 and Serotonin
0.040462	Excitatory Neural Signalling Through 5-HTR 7 and Serotonin
0.040462	Excitatory Neural Signalling Through 5-HTR 4 and Serotonin
0.043495	cGMP-PKG signaling pathway - Homo sapiens (human)
0.043495	Antigen processing and presentation - Homo sapiens (human)
0.046476	B cell receptor signaling pathway - Homo sapiens (human)
0.046476	Intracellular Signalling Through LHCGR Receptor and Luteinizing Hormone/Choriogonadotropin
0.046476	Intracellular Signalling Through FSH Receptor and Follicle Stimulating Hormone
0.062064	protein kinase a at the centrosome
0.062064	Opioid Signalling
0.062064	activation of csk by camp-dependent protein kinase inhibits signaling through the t cell receptor
0.062064	CLEC7A (Dectin-1) induces NFAT activation
0.062064	attenuation of gpcr signaling
0.066339	protein ubiquitylation
0.071469	Metabolism of lipids and lipoproteins
0.073139	mRNA decay by 5, to 3, exoribonuclease
0.075254	Deadenylation-dependent mRNA decay
0.085876	PI Metabolism
0.090806	Phosphorylation of CD3 and TCR zeta chains
0.090806	Herpes simplex infection - Homo sapiens (human)
0.090806	MHC class II antigen presentation

Table S6. Relevant features from the survival analysis for females (A) and males (B)

A)

Gene symbol	Agilent ID	Active.Coefficients
SMCR7L	A_23_P109547	-0.04245
NT5E	A_23_P111260	-0.01521
KLRC3	A_23_P128281	-0.09766
SS18	A_23_P141738	0.087289

WDR4	A_23_P143535	0.017689
TGIF1	A_23_P153197	0.03227
API5	A_23_P203255	-0.02204
KIAA0408	A_23_P215048	0.042344
C10orf28	A_23_P301336	-0.00318
KCNV2	A_23_P32083	0.001033
C20orf11	A_23_P56971	0.121936
HIST1H3F	A_23_P8004	0.055795
KDM2B	A_23_P87919	0.060316
KYNU	A_24_P11506	0.065493
RAVER1	A_24_P126557	-0.07503
	A_24_P144163	-0.161
	A_24_P178654	-0.02931
	A_24_P605563	-0.00492
	A_24_P67432	-0.00259
RRP7B	A_24_P83158	0.012215
JAM3	A_24_P86993	-0.00355
ARHGEF6	A_24_P926566	-0.00554
SMAD2	A_32_P109002	0.094398
	A_32_P86616	0.040808

B)

Gene symbol	Agilent ID	Active.Coefficients
MFHAS1	A_23_P112078	0.037131
ZNF8	A_23_P130470	0.00185
NLE1	A_23_P141315	-0.15334
DUSP26	A_23_P146134	0.007499
TRMT1	A_23_P16683	-0.03086
KCNH2	A_23_P168403	0.088035
GFM2	A_23_P213431	0.290748
ENTPD4	A_23_P216017	0.027358
LRRN3	A_23_P31376	-0.0486
C1orf109	A_23_P45970	-0.26535
GLYATL2	A_23_P47484	0.075721
LANCL2	A_23_P82642	0.108439
DEPDC5	A_23_P91680	0.004139
0	A_24_P135933	-0.07269
DTNB	A_24_P216421	-0.02668
IGLL1	A_24_P239076	-0.0409
NT5E	A_24_P354715	-0.01442
0	A_24_P476386	0.099122
0	A_24_P484904	-0.00082

0	A_24_P639701	-0.00541
0	A_24_P683861	-0.19698
FAM90A7	A_24_P903680	0.037586
C14orf132	A_24_P920447	0.024199
RASA4	A_24_P943263	0.142848
ANXA8L2	A_32_P105549	0.11858
0	A_32_P108554	-0.05101
0	A_32_P182609	0.006721
RPS15A	A_32_P80901	-0.11653

Table S7. Pathways analysis from the features highlighted in Table S6.

A)

p-value	q-value	pathway
2.86E-05	0.000686	Tgif disruption of Shh signaling
0.000166	0.001988	Downregulation of SMAD2/3:SMAD4 transcriptional activity
0.00055	0.004398	Transcriptional activity of SMAD2/SMAD3:SMAD4 heterotrimer
0.00099	0.005525	HDMs demethylate histones
0.001151	0.005525	TGF-beta Receptor Signaling
0.001703	0.006727	Signaling by TGF-beta Receptor Complex
0.002129	0.006727	Pancreatic cancer - Homo sapiens (human)
0.002242	0.006727	Regulation of nuclear SMAD2/3 signaling
0.002662	0.007098	TGF-beta signaling pathway - Homo sapiens (human)
0.00346	0.008305	Signaling by TGF-beta family members
0.00644	0.014051	TGF-beta Signaling Pathway

B)

p-value	q-value	pathway
0.000357	0.003167	Nucleobase catabolism
0.000555	0.003167	Purine metabolism
0.000679	0.003167	Pyrimidine nucleotides nucleosides metabolism
0.002633	0.006882	Pyrimidine metabolism - Homo sapiens (human)
0.002842	0.006882	Metabolism of nucleotides
0.002949	0.006882	Purine nucleotides nucleosides metabolism
0.00465	0.0093	Pyrimidine metabolism
0.007529	0.013176	Purine metabolism - Homo sapiens (human)

Figure S2. ROC curves from the future lymphoma predictive models

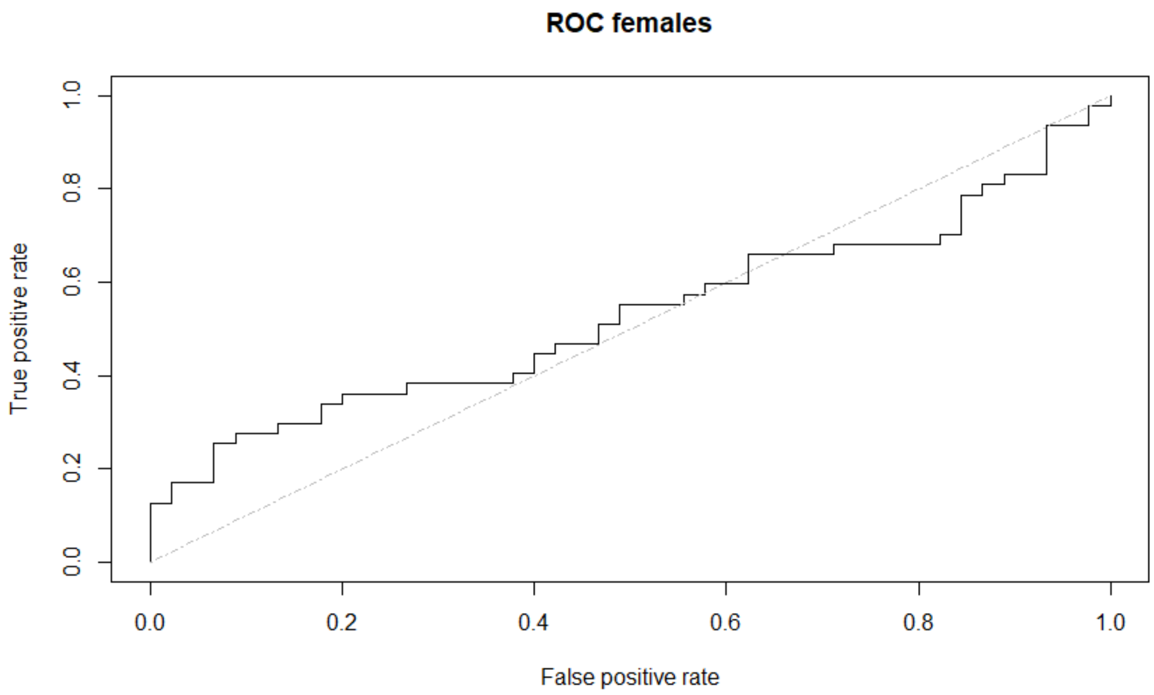

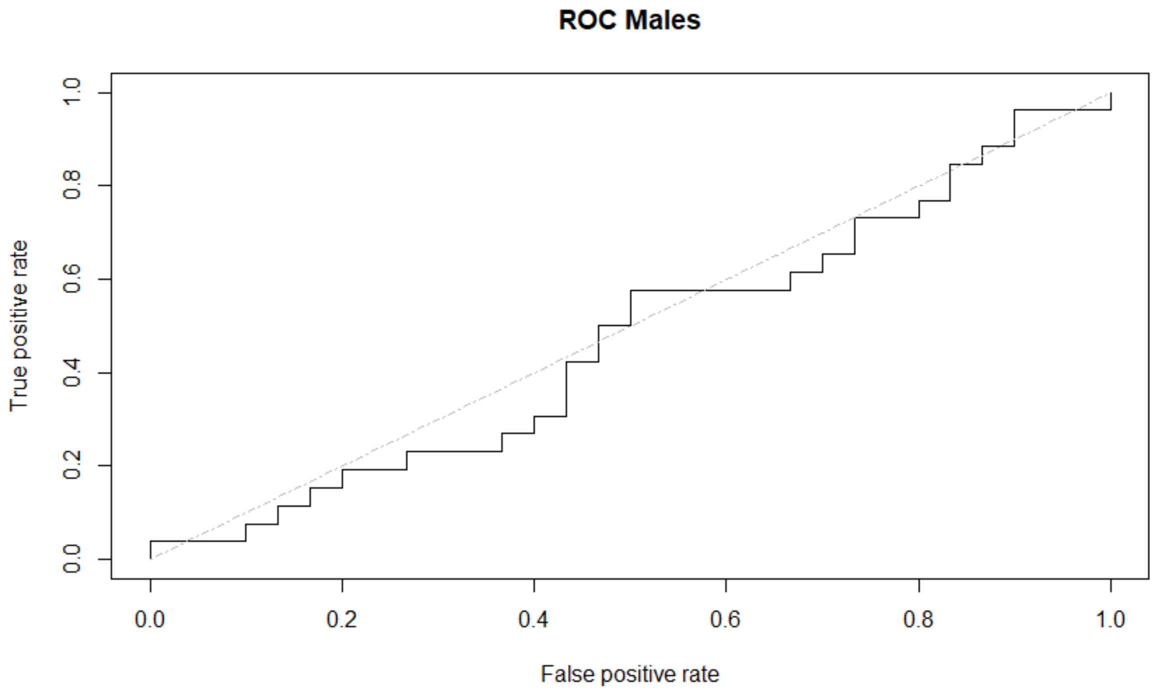

Table S8. Overlaps between the top 5% genes of the lymphoma profile and LMM (FDR<0.05).

Population	Cell type	PCB	Overlap top 5% up	Overlap top 5% down	LMM (LY Future cases)	% overlap up LMM	% overlap down LMM	% overlap up profile	% overlap down profile
Females	B cells	118	18	17	1055	1.71	1.61	1.8	1.7
Females	B cells	138	15	0	650	2.31	0.00	1.5	0
Females	B cells	153	14	0	563	2.49	0.00	1.4	0
Females	B cells	156	0	0	48	0.00	0.00	0	0
Females	B cells	170	0	0	14	0.00	0.00	0	0
Females	B cells	180	1	0	127	0.79	0.00	0.1	0
Females	CD8T	118	0	0	0	0.00	0.00	0	0
Females	CD8T	138	0	0	1	0.00	0.00	0	0
Females	CD8T	153	0	0	5	0.00	0.00	0	0
Females	CD8T	156	0	0	4	0.00	0.00	0	0
Females	CD8T	170	0	0	4	0.00	0.00	0	0
Females	CD8T	180	0	0	3	0.00	0.00	0	0
Females	CD4T	118	5	81	3121	0.16	2.60	0.5	8.1
Females	CD4T	138	3	62	2755	0.11	2.25	0.3	6.2
Females	CD4T	153	5	70	3004	0.17	2.33	0.5	7
Females	CD4T	156	7	95	3586	0.20	2.65	0.7	9.5
Females	CD4T	170	10	88	4326	0.23	2.03	1	8.8
Females	CD4T	180	12	97	5315	0.23	1.83	1.2	9.7
Females	NK	118	0	0	2	0.00	0.00	0	0
Females	NK	138	0	0	1	0.00	0.00	0	0
Females	NK	153	0	0	1	0.00	0.00	0	0
Females	NK	156	0	3	73	0.00	4.11	0	0.3
Females	NK	170	0	1	21	0.00	4.76	0	0.1
Females	NK	180	0	0	1	0.00	0.00	0	0
Females	Mono	118	4	47	723	0.55	23.24	0.4	4.7
Females	Mono	138	26	116	7445	0.35	1.56	2.6	11.6
Females	Mono	153	27	126	8373	0.32	1.50	2.7	12.6
Females	Mono	156	30	168	10120	0.30	1.66	3	16.8
Females	Mono	170	26	149	9352	0.28	1.59	2.6	14.9
Females	Mono	180	27	115	7944	0.34	1.45	2.7	11.5
Males	B cells	118	19	1	238	7.98	0.42	1.9	0.1
Males	B cells	138	13	1	195	1.03	0.51	0.2	0.1
Males	B cells	153	2	1	61	3.28	1.64	0.2	0.1
Males	B cells	156	3	0	28	0	0	0	0
Males	B cells	170	2	0	24	0	0	0	0
Males	B cells	180	2	1	14	0	0	0	0
Males	CD8T	118	0	0	0	0	0	0	0
Males	CD8T	138	0	0	0	0	0	0	0
Males	CD8T	153	0	0	0	0	0	0	0
Males	CD8T	156	0	0	0	0	0	0	0
Males	CD8T	170	0	0	0	0	0	0	0
Males	CD8T	180	0	0	0	0	0	0	0
Males	CD4T	118	0	0	0	0	0	0	0
Males	CD4T	138	0	0	0	0	0	0	0
Males	CD4T	153	0	0	0	0	0	0	0
Males	CD4T	156	0	0	0	0	0	0	0
Males	CD4T	170	0	0	0	0	0	0	0
Males	CD4T	180	0	0	0	0	0	0	0
Males	NK	118	0	0	0	0	0	0	0
Males	NK	138	0	0	0	0	0	0	0
Males	NK	153	0	0	0	0	0	0	0
Males	NK	156	0	0	1	0	0	0	0
Males	NK	170	0	0	0	0	0	0	0
Males	NK	180	0	0	0	0	0	0	0
Males	Mono	118	0	0	1	0	0	0	0
Males	Mono	138	0	0	2	0	0	0	0
Males	Mono	153	0	0	0	0	0	0	0
Males	Mono	156	0	0	0	0	0	0	0
Males	Mono	170	0	0	5	0	0	0	0
Males	Mono	180	0	0	1	0	0	0	0

Table S9. 150 unique pathways from the genes overlapping with the lymphoma profile.

List of pathways
Glycosaminoglycan metabolism
Separation of Sister Chromatids
Mitotic Anaphase
Mitotic Metaphase and Anaphase
Chemokine receptors bind chemokines
Chemokine signaling pathway - Homo sapiens (human)
Peptide ligand-binding receptors
G alpha (i) signalling events
Immunoregulatory interactions between a Lymphoid and a non-Lymphoid cell
Platelet activation, signaling and aggregation
Class A/1 (Rhodopsin-like receptors)
Cytokine-cytokine receptor interaction - Homo sapiens (human)
GPCR ligand binding
Hemostasis
mcalpain and friends in cell motility
Platelet degranulation
Sodium/Calcium exchangers
Response to elevated platelet cytosolic Ca2+
Graft-versus-host disease - Homo sapiens (human)
Formation of Fibrin Clot (Clotting Cascade)
Natural killer cell mediated cytotoxicity - Homo sapiens (human)
Eicosanoid Synthesis
Common Pathway of Fibrin Clot Formation
ras-independent pathway in nk cell-mediated cytotoxicity
Prostaglandin Leukotriene metabolism
stathmin and breast cancer resistance to antimicrotubule agents
Opioid Signalling
Ketoprofen Action Pathway
Acetylsalicylic Acid Action Pathway
Diflunisal Action Pathway
Acetaminophen Action Pathway
Sulindac Action Pathway
Ketorolac Action Pathway
Naproxen Action Pathway
Flurbiprofen Action Pathway
Antrafenine Action Pathway
Trisalicylate-choline Action Pathway
Nepafenac Action Pathway
Phenylbutazone Action Pathway
Lornoxicam Action Pathway
Salsalate Action Pathway
Salicylic Acid Action Pathway
Salicylate-sodium Action Pathway

Oxaprozin Action Pathway
Nabumetone Action Pathway
Bromfenac Action Pathway
Mefenamic Acid Action Pathway
Piroxicam Action Pathway
Carprofen Action Pathway
Fenoprofen Action Pathway
Antipyrine Action Pathway
Magnesium salicylate Action Pathway
Tenoxicam Action Pathway
Tiaprofenic Acid Action Pathway
Tolmetin Action Pathway
Suprofen Action Pathway
Etodolac Action Pathway
Rofecoxib Action Pathway
Diclofenac Action Pathway
Etoricoxib Action Pathway
Lumiracoxib Action Pathway
Valdecoxib Action Pathway
Meloxicam Action Pathway
Leukotriene C4 Synthesis Deficiency
Arachidonic Acid Metabolism
GPCR downstream signaling
G-protein activation
Indomethacin Action Pathway
Smooth Muscle Contraction
erk and pi-3 kinase are necessary for collagen binding in corneal epithelia
G Protein Signaling Pathways
Vindesine Action Pathway
Vincristine Action Pathway
Vinblastine Action Pathway
Vinorelbine Action Pathway
Etoposide Pathway, Pharmacokinetics/Pharmacodynamics
Etoposide Action Pathway
Etoposide Metabolism Pathway
Glucagon signaling in metabolic regulation
Glucagon-like Peptide-1 (GLP1) regulates insulin secretion
Vasopressin regulates renal water homeostasis via Aquaporins
Calcium Regulation in the Cardiac Cell
G alpha (z) signalling events
PLC beta mediated events
Aquaporin-mediated transport
G-protein mediated events
IL12-mediated signaling events
Platelet Aggregation Inhibitor Pathway, Pharmacodynamics
Platelet activation - Homo sapiens (human)

Syndecan interactions
eicosanoid metabolism
Thromboxane signalling through TP receptor
ECM-receptor interaction - Homo sapiens (human)
Molecules associated with elastic fibres
Signaling by GPCR
Signal amplification
Celecoxib Action Pathway
Prostaglandin formation from arachidonate
Elastic fibre formation
Serotonergic synapse - Homo sapiens (human)
Platelet Aggregation (Plug Formation)
CXCR3-mediated signaling events
BMP receptor signaling
Non-integrin membrane-ECM interactions
Hematopoietic cell lineage - Homo sapiens (human)
Antigen processing and presentation - Homo sapiens (human)
Cell surface interactions at the vascular wall
Phagosome - Homo sapiens (human)
ucalpain and friends in cell spread
integrin signaling pathway
HDMs demethylate histones
RhoA signaling pathway
Cell Cycle, Mitotic
agrin in postsynaptic differentiation
Signaling events mediated by focal adhesion kinase
Cell Cycle
G1 to S cell cycle control
AP-1 transcription factor network
SUMOylation of DNA damage response and repair proteins
SUMO E3 ligases SUMOylate target proteins
Mitotic G1-G1/S phases
SUMOylation
RHO GTPase Effectors
Senescence-Associated Secretory Phenotype (SASP)
G2/M Checkpoints
Oxidative Stress Induced Senescence
Endothelin Pathways
Adaptive Immune System
Human Complement System
Immune System
G alpha (q) signalling events
pkc-catalyzed phosphorylation of inhibitory phosphoprotein of myosin phosphatase
Muscle contraction
RHO GTPases activate PAKs
Regulation of retinoblastoma protein

Small Ligand GPCRs
GPCRs, Class A Rhodopsin-like
Gastrin-CREB signalling pathway via PKC and MAPK
Ethanol oxidation
RHO GTPases activate PKNs
Ifosfamide Pathway, Pharmacodynamics
Fatty Acid Omega Oxidation
Myometrial Relaxation and Contraction Pathways
Cyclophosphamide Pathway, Pharmacodynamics
Prostacyclin signalling through prostacyclin receptor
Downstream signaling in naïve CD8+ T cells
G beta:gamma signalling through PLC beta
Post-translational protein modification
Cell cycle - Homo sapiens (human)
